# Chitosan Oligosaccharide Prevents Afatinib-Induced Barrier Disruption and Chloride Secretion through Modulation of AMPK, PI3K/AKT, and ERK Signaling in T84 Cells

**DOI:** 10.3390/polym14204255

**Published:** 2022-10-11

**Authors:** Tahir Mehmood, Rath Pichyangkura, Chatchai Muanprasat

**Affiliations:** 1 Chakri Naruebodindra Medical Institute, Faculty of Medicine Ramathibodi Hospital, Mahidol University, Bang Phli, Samut Prakan 10540, Thailand; 2 Department of Biochemistry, Faculty of Science, Chulalongkorn University, Patumwan, Bangkok 10400, Thailand

**Keywords:** chitosan oligosaccharide, afatinib, epidermal growth factor receptors, EGFR-tyrosine kinase inhibitors, diarrhea

## Abstract

Diarrhea is an important adverse effect of epidermal growth factor receptor-tyrosine kinase inhibitors, especially afatinib. Novel antidiarrheal agents are needed to reduce epidermal growth factor receptor-tyrosine kinase inhibitor-associated diarrhea to improve the quality of life and treatment outcome in cancer patients. This study aimed to investigate the anti-diarrheal activity of chitosan oligosaccharide against afatinib-induced barrier disruption and chloride secretion in human intestinal epithelial cells (T84 cells). Chitosan oligosaccharide (100 μg/mL) prevented afatinib-induced barrier disruption determined by changes in transepithelial electrical resistance and FITC-dextran flux in the T84 cell monolayers. In addition, chitosan oligosaccharide prevented afatinib-induced potentiation of cAMP-induced chloride secretion measured by short-circuit current analyses in the T84 cell monolayers. Chitosan oligosaccharide induced the activation of AMPK, a positive regulator of epithelial tight junction and a negative regulator of cAMP-induced chloride secretion. Moreover, chitosan oligosaccharide partially reversed afatinib-induced AKT inhibition without affecting afatinib-induced ERK inhibition via AMPK-independent mechanisms. Collectively, this study reveals that chitosan oligosaccharide prevents the afatinib-induced diarrheal activities in T84 cells via both AMPK-dependent and AMPK-independent mechanisms. Chitosan oligosaccharide represents a promising natural polymer-derived compound for further development of treatment for afatinib-associated diarrheas.

## 1. Introduction

The epidermal growth factor receptors (EGFRs), a family of receptor tyrosine kinases (RTKs), are the most extensively studied drug targets for many tumors as they are aberrantly expressed in cancer cells and are actively involved in cell proliferation and differentiation [1,2]. Several small molecule EGFR-tyrosine kinase inhibitors (TKIs) have been developed for the treatment of cancers overexpressing EGFRs including breast cancer, head and neck cancer, and non-small cell lung cancer [1,3,4,5,6]. However, these EGFR-TKIs induce an on target-off tumor effect since normal cells including intestinal epithelial cells also express EGFRs. This on target-off tumor effect causes diarrhea by modifying intestinal fluid transport mechanisms. Diarrhea induction leads to treatment discontinuation and dose reduction, which reduces the therapeutic outcomes of EGFR-TKIs. The severity of EGFR-TKI-associated diarrheas (grades 1–4) differs among different types of EGFR-TKIs. First generation EGFR-TKIs are less brutal, with 10–20% of patients developing diarrhea of grades 3–4. Second generation EGFR-TKIs including afatinib develop severe diarrhea (grades 3–4) in more than 90% of patients [7,8,9].

Intestinal epithelial cells produce active chloride ion (Cl^−^) secretion to maintain intestinal fluid balance across the intestinal epithelial barriers. Cl^−^ enters into the intestinal epithelial cells by Na^+^-K^+^-2Cl^−^–cotransporter1 (NKCC1) on the basolateral membrane and exits to the intestinal lumen via cystic fibrosis transmembrane conductance regulator (CFTR) and calcium-dependent chloride channels (CaCC) [7,10,11,12]. An adenosine 3′,5′-cyclic monophosphate (cAMP)-dependent pathway stimulates CFTR and a calcium-dependent pathway stimulates CaCC to induce the prolonged and transient Cl^−^ secretory responses, respectively [10,11,12]. Na^+^/K^+^ pumps on basolateral membrane of the cell along with basolateral K^+^ channels provide the driving force for Cl^−^ secretion by maintaining negative membrane potential required for driving Cl^−^ efflux across the apical membrane [12,13]. These processes are precisely regulated and disturbance in this complex network may lead to excessive Cl^−^ secretion, resulting in diarrhea. Conventional chemotherapeutic agents directly damage epithelial cells resulting in an onset of diarrhea. Interestingly, EGFR-TKI inhibitors such as afatinib exhibit diarrheal activity through the induction of dysfunctions in epithelial ion transport or barrier function, inflammation, and mucosal injury [14]. Previous studies in T84 cells have demonstrated that afatinib potentiated calcium-induced Cl^−^ secretion by the stimulation of CFTR and basolateral K^+^ channels [7].

Chitosan oligosaccharide (COS) is derived by the hydrolysis and deacetylation of chitin, a polymer of N-acetyl-D-glucosamine, which is a main constituent of the exoskeleton of arthropods such as insects, crabs and shrimps, and the cell wall of fungi [15,16,17,18,19]. The physico-chemical and biological properties of this biopolymer have been explored in various fields including pharmaceuticals, biotechnology, textiles, cosmetics, biomedical engineering, agriculture, food processing, nutrition, water, and wastewater treatments [20,21,22,23]. Due to its favorable pharmacokinetics and beneficial biological activities including anti-oxidative, anti-inflammatory, anti-diabetic, anti-bacterial, and anti-cancer activities, COS has the potential for development as dietary supplements or nutraceuticals [15,16,17,18,19,20,21,22,23,24,25,26,27]. Previously, we have reported that COS activated AMP-activated protein kinase (AMPK) in the intestinal epithelial cells via a mechanism involving calcium sensing receptor (CaSR)-mediated calcium release from the endoplasmic reticulum. COS enhanced the tight junction assembly in the T84 cell monolayers in an AMPK- and extracellular Ca^2+^-dependent manner [28]. Furthermore, COS exerts a chemopreventive effect in colorectal cancer mouse models via AMPK activation and NF-кB suppression [29]. The effect of COS on afatinib-induced intestinal toxicities remains unknown. Unlike conventional chemotherapeutic drugs that directly damage epithelial cells, afatinib exhibits diarrheal activity through barrier integrity disruption and inducing epithelial ion secretion [14]. Afatinib potentiates calcium-induced Cl^−^ secretion by the stimulation of CFTR and basolateral K^+^ channels [7]. In addition, afatinib inhibits EGFR-mediated PI3K/AKT/mTOR signaling in vitro and in vivo in neuroblastoma cells [30] and inhibits tumorigenesis in glioblastoma by inhibiting EGFR/AKT signaling [31]. Inhibition of AKT induces Cl^−^ secretion, leading to the development of diarrheas [32]. Therefore, the present study aimed to investigate the effect of COS on afatinib-induced abnormal intestinal functions involved in the pathogenesis of TKI-associated diarrheas including intestinal barrier disruption and Cl^−^ secretion and its underlying mechanisms using a human intestinal epithelial cell line (T84 cells).

## 2. Materials and Methods

### 2.1. Materials

T84 cells were obtained from the American Type Culture Collection (Manassas, VA, USA). DMEM/Ham’s F-12 media, Minimum Essential Medium Eagle, Spinner Modification (S-MEM), penicillin, and streptomycin were purchased from Gibco, ThermoFisher Scientific (Waltham, MA, USA). MTT was purchased from Bio Basic Inc. (Markham, Canada). DMSO, FITC-dextran (molecular weight of 4400 Da,) and forskolin were purchased from Sigma Aldrich (St. Louis, MO, USA). Afatinib, CFTR-172, and Compound C were purchased from Merck Millipore (Darmstadt, Germany). Antibodies including AMPK-α, p-AMPK-α, ACC, p-ACC, AKT, p-AKT, ERK42/44, p-ERK42/44, and β-actin were purchased from Cell Signaling Technology (Danvers, MA, USA).

### 2.2. Cell Culture

T84 cells were cultured in 1:1 Dulbecco’s modified Eagle’s medium (DMEM) and Ham’s F-12 medium supplemented with 10% FBS, 100 U/mL penicillin, and 100 mg/mL streptomycin. Cells were maintained at 37 °C with 5% CO_2_/95% O_2_ in a humidified atmosphere.

### 2.3. Cell Viability Assays

The effect of afatinib and COS on the viability of T84 cells was determined by the MTT assay, as described previously [33]. Briefly, 5000 cells of T84 cells were seeded and cultured in 96-well cell culture plates for 24 h at 37 °C. Cells were treated with different concentrations of afatinib and COS either alone or in combination for 24 h. Following treatment, cells were incubated with 10 µL MTT (5 mg/mL) reagent at 37 °C for 4 h. Subsequently, 150 µL DMSO was added to dissolve formazan crystals and absorbance was measured at 570 nm by a Synergy/neo2 multi-mode reader. The data are presented as the percentage of cell viability compared to the control.

### 2.4. Measurement of Tight Junction Assembly

The measurement of the integrity of the epithelial tight junction was determined with or without the Ca^2+^ switch assay, as previously described [28]. For the Ca^2+^ switch assays, T84 cells were seeded on a Snapwell permeable support (5 × 10^5^ cells/support) and cultured for 3 days. Media were changed every 48 h. To assess the monolayer integrity, an EVOM2 voltohm meter (World Precision Instruments, Inc., Sarasota, FL, USA) with a chopstick electrode set was used to measure the transepithelial electrical resistance (TEER) across the monolayer. The monolayers with TEER more than 1000 Ω·cm^2^ for two consecutive days were used in this experiment. Then, DMEM/Ham’s F12 culture media were removed and replaced with Ca^2+^-free Minimum Essential Medium Eagle, Spinner Modification (S-MEM) for 16 h to disrupt the tight junctions. Subsequently, Ca^2+^ switch was performed by replacing S-MEM media with regular DMEM/ham’s F-12 containing Ca^2+^ supplemented with the vehicle, afatinib (10 µM), COS (100 μg/mL), or COS (100 μg/mL) plus afatinib (10 µM). TEER was measured before and after Ca^2+^ switch at different time intervals. In some experiments, TEER was measured with different treatments of COS and afatinib without Ca^2+^ switch.

### 2.5. FITC-Dextran Flux Assay

The measurement of integrity of the epithelial tight junction was performed by the FITC-dextran flux assay, as previously described [28]. T84 cells were seeded on a Transwell permeable support and cultured for 7 days to develop monolayers. T84 cell monolayers were treated with the vehicle, afatinib (10 µM), or COS (100 µg/mL) either alone or in combination for 24 h. Following treatment, FITC-dextran was added into the apical media (1 mg/mL) and, one and a half hours later, basolateral media were sampled for the determination of FITC-dextran concentrations using a Synergy/neo2 multi-mode reader.

### 2.6. Short-Circuit Current Measurement

The short-circuit current (I_SC_) was measured as previously described [34,35]. Briefly, T84 cell monolayers were mounted in Ussing chambers (Physiologic Instruments, Reno, NV, USA) and bathed bilaterally with 5 mL oxygenated (95% O_2_, 5% CO_2_) Kreb’s solution at 37 °C. The composition of the Kreb’s solution was: 120 mM NaCl, 25 mM NaHCO_3_, 0.8 mM K_2_HPO_4_, 3.3 mM KH_2_PO_4_, 1.2 mM MgCl_2_, and 1.2 mM CaCl_2_. The short-circuit current (I_SC_) was measured using a DVC-1000 voltage-clamp with Ag/AgCl electrodes and 3 M KCl agar bridges.

### 2.7. Western Blot Analysis

Proteins extracts for Western blot analysis were prepared as previously described [33]. Briefly, T84 cells were treated with different concentrations of afatinib or COS either alone or in combination in the presence or absence of an AMPK inhibitor, Compound C (40 µM) for 24 h, and lysed on ice with RIPA cell lysis reagent supplemented with 1% phosSTOP and protease inhibitors (Mannhein, Germany). Protein concentrations in the cell lysates were determined by the Bradford reagent using the Lowry method. Twenty five micrograms of proteins were resolved on 10% sodium dodecyl sulfate-polyacrylamide gel electrophoresis and transferred to a nitrocellulose membrane. Membranes were blocked with 5% (*w/v*) nonfat milk for 1 h and incubated overnight at 4 °C with antibodies to AMPK-α (1:1000), p-AMPK-α (1:1000), ACC (1:1000), p-ACC (1:1000), AKT (1:1000), p-AKT (1:1000), ERK42/44 (1:1000), p-ERK42/44 (1:1000), or β actin (1:1000). After washing with TBST, the blots were incubated with horseradish peroxidase-conjugated goat anti-rabbit second antibodies for an hour at room temperature. After washing with TBST, signals were detected using the ECL Plus Chemiluminescence Kit by Bio-Rad ChemiDocTM Imaging System. Densitometry analysis was performed by ImageJ software and presented in graphical format.

### 2.8. Statistics

Data were presented as mean ± S.D. from at least three different independent experiments and were statistically compared with the untreated control group or compared within the treated groups using a repeated measures analysis of variance (ANOVA) followed by Tukey’s post hoc test, by GraphPad Prism software. Furthermore, *p*-values < 0.05 were considered statistically significant. Columns not sharing the same superscript letters were statistically significant.

## 3. Results

### 3.1. COS Promotes Tight Junction Assembly

**Figure 1:**
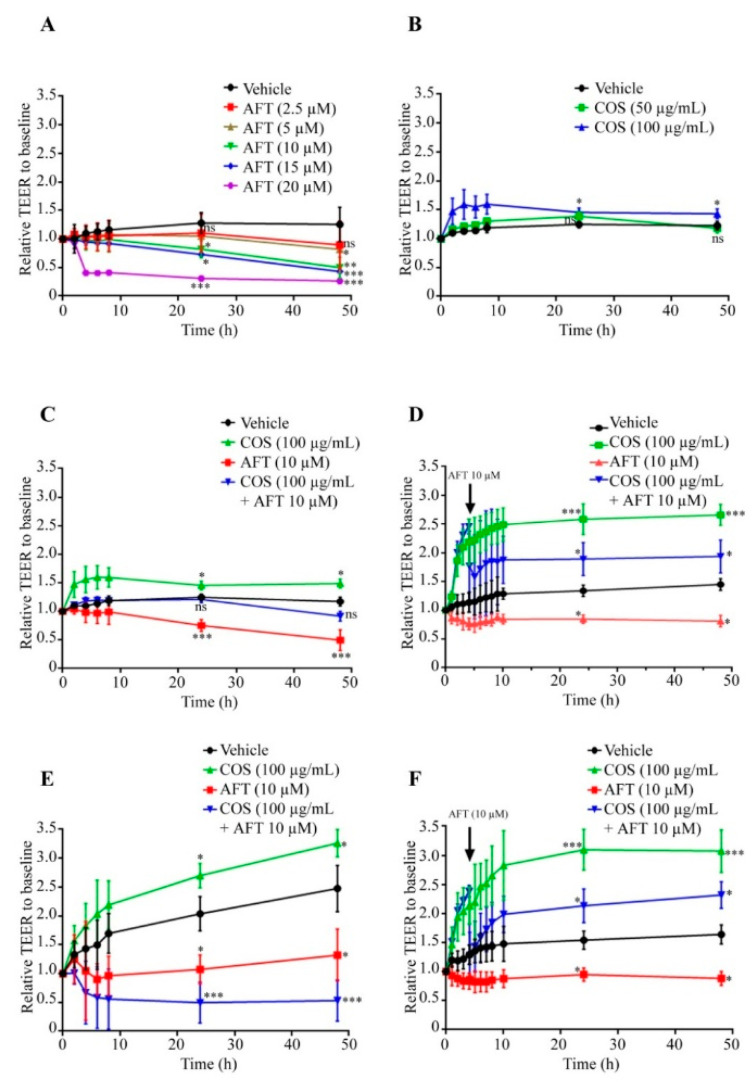
Effect of afatinib and COS on the tight junction assembly. TEER across T84 cell monolayers was measured at different time intervals after incubation with the indicated concentrations of afatinib (**A**) and COS (**B**). (**C**) The effect of afatinib and COS either alone or in combination on tight junction assembly. (**D**) Preventive effect of COS on afatinib-induced tight junction disruption. (**E**) Tight junction assembly measurements using Ca^2+^ switch assays. T84 cell monolayers were exposed to Ca^2+^ free media. After 16 h, Ca^2+^ free media were replaced with normal media containing afatinib and COS followed by TEER measurement across T84 cell monolayers. (**F**) Preventive effect of COS on afatinib-induced tight junction disruption measured by Ca^2+^ switch assays. Data are expressed as the means of TEER ± SD (*n* = 5) (one-way ANOVA; * *p* < 0.05; ** *p* < 0.01; *** *p* < 0.001 compared with vehicle treated group).

As afatinib is known to induce diarrhea by both disrupting the epithelial barrier and inducing Cl^−^ secretion [14], we determined the effect of afatinib on the tight junction integrity in the T84 cell monolayers. The data showed that afatinib significantly decreased TEER in a concentration-dependent manner compared to the vehicle-treated group (control), as shown in Figure 1A. Since it was established that COS accelerated tight junction assembly [28], we measured the effect of COS (100 µg/mL) on the tight junction integrity in the T84 cell monolayers. As depicted in Figure 1B, COS significantly increased TEER in a concentration-dependent manner compared to the control. The TEER in the COS-treated groups dropped to the control level at 48 h post-treatment. In the cotreatment experiments, COS reversed the inhibitory effect of afatinib on TEER. After 24 h of post-treatment, the TEER slightly decreased in cotreatment conditions, as shown in Figure 1C. These findings suggest that COS promoted tight junction integrity. To evaluate the preventive effect of COS on afatinib-induced tight junction disruption, cells were pre-incubated with COS for 4 h followed by afatinib treatment. Figure 1D depicted that TEER decreased immediately and started to increase after 30 min of afatinib treatment. Pretreatment with COS prevented the inhibitory effect of afatinib on TEER above the control group, indicating that COS prevented the afatinib-induced loss of tight junction integrity.

**Figure 2:**
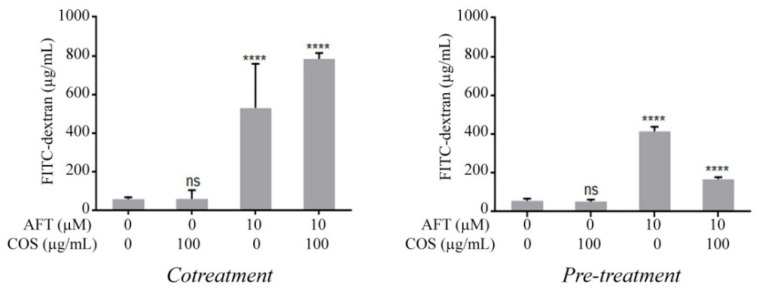
Effect of afatinib and COS on barrier junction. (*Cotreatment*) T84 cell monolayers were treated with the indicated concentrations of reagents for 24 h. (*Pre-treatment*) Cells was pre-incubated with COS for 4 h followed by incubation with afatinib. Data are expressed as means of FITC-dextran concentration ± SD. (*n* = 5; **** *p* < 0.0001; one-way ANOVA).

Previously, we have reported that COS enhanced the extracellular Ca^2+^-induced tight junction assembly. Next, we explored the effect of COS on extracellular Ca^2+^-induced tight junction assembly either alone or in combination with afatinib in the T84 cell monolayers using Ca^2+^ switch assays. COS significantly increased TEER compared with the control. These data are in agreement with our previous report [28]. Afatinib decreased TEER, which was further decreased by COS cotreatment (Figure 1E). We hypothesized that COS required incubation periods to exert a protective effect on the tight junction. We next performed a 4-h pre-incubation with COS followed by afatinib treatment and measurements of the Ca^2+^ switch assays. As depicted in Figure 1F, the afatinib-induced TEER decrement was recovered by COS pretreatment to the level that was above the control group. Furthermore, the effect of COS and afatinib on the tight junction integrity was determined by FITC-dextran (molecular weight of 4.4 kDa) flux assays. COS did not affect the FITC-dextran flux compared to the control (Figure 2). Afatinib induced FITC-dextran flux, which was unaffected by cotreatment with COS. We next investigated the effect of 4-h pretreatment with COS and found that COS significantly reduced the afatinib-induced FITC-dextran flux. These data suggested that COS promoted extracellular Ca^2+^-induced tight junction assembly and prevented the deleterious effect of afatinib on the tight junction integrity in the T84 cell monolayers.

### 3.2. Effect of COS on Afatinib-Induced Cl^−^ Secretion across T84 Cell Monolayers

**Figure 3:**
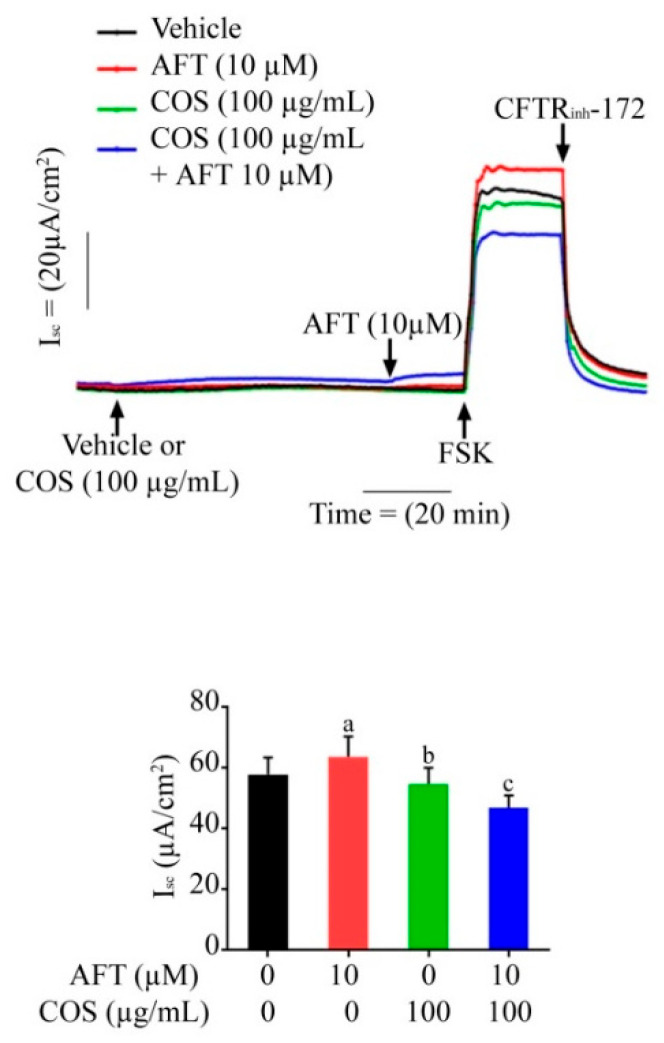
Effect of COS on afatinib-induced Cl^−^ secretion. T84 cell monolayers were mounted in Ussing chambers and treated with the vehicle control, COS, or afatinib alone or in combination. Chloride secretion was then induced with forskolin and quantified as the change in short circuit current (ΔI_sc_). Data are expressed as means of I_sc_ ± SD. (*n* = 5; *p* < 0.05; one-way ANOVA). Columns not sharing the same superscript letters differ significantly.

The effect of afatinib on cAMP-induced Cl^−^ secretion was determined in the T84 cell monolayers using I_sc_ measurements. We found that afanitib pretreatment significantly increased the cAMP-induced Cl^−^ secretion stimulated by forskolin, a cAMP agonist. COS pretreatment completely abolished the cAMP-induced Cl^−^ secretion potentiated by afatinib (Figure 3). Of note, COS pretreatment slightly inhibited cAMP-induced Cl^−^ secretion in the absence of afatinib. Indeed, we found no effect of afatinib on potentiating carbachol-induced Cl^−^ secretion in T84 cells.

### 3.3. Effect of COS and Afatinib on AMPK Signaling

**Figure 4:**
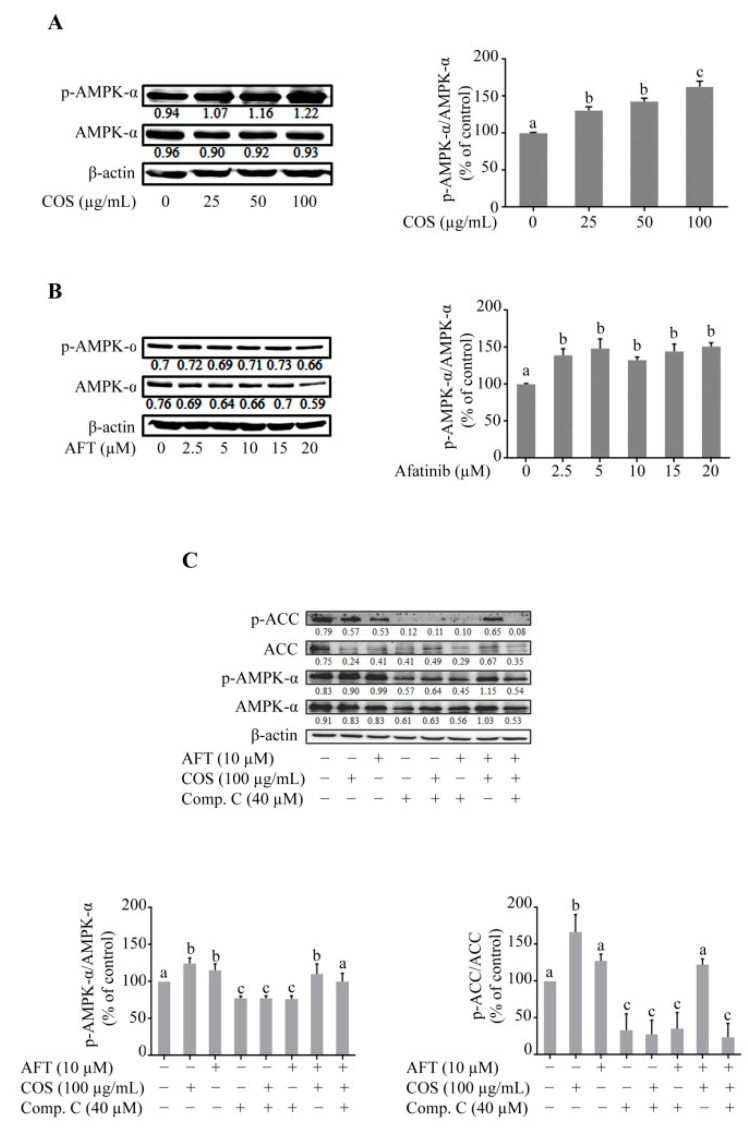
The effects of COS and afatinib on AMPK activation. T84 cells were treated with the indicated concentrations of COS (**A**), afatinib (**B**) or in combination (**C**) in the presence or absence of an AMPK inhibitor, Compound C (40 µM), for 24 h before performing Western blot analysis. Data were analyzed as the ratio of p-AMPK-α/AMPK-α and p-ACC/ACC and expressed as % of control (vehicle-treated group), means ± SD (*n* = 5; *p* < 0.05 one-way ANOVA). Columns that do not share the same superscript letters differ significantly (*p* < 0.05, Tukey’s post hoc test).

Accumulating evidence suggests that COS-induced tight junction assembly and the inhibition of Cl^−^ secretion in epithelial cells are mediated by AMPK [28]. We determined the role of AMPK activation in T84 cells after afatinib and COS treatment by Western blot analyses. Figure 4A shows that the expression level of total AMPK-α was not changed in the COS-treated groups. COS significantly increased the phosphorylation of AMPK-α and increased the ratio of p-AMPK-α/AMPK-α in a concentration-dependent manner. In the afatinib-treated groups, the phosphorylated form of AMPK-α was not increased whereas the expression level of total AMPK-α was decreased, resulting in an increase in the ratio of p-AMPK-α/AMPK-α compared to the vehicle, as shown in Figure 4B. Furthermore, the effect of COS on AMPK activation and its downstream target protein, acetyl co-A carboxylase (ACC), either alone or in combination with afatinib, in the presence or absence of Compound C (40 µM), an AMPK inhibitor, was determined by Western blot analysis. COS significantly increased the expression level of p-AMPK-α and p-ACC and increased the ratio of p-AMPK-α/AMPK-α and p-ACC/ACC, whereas afatinib did not produce significant effects on the p-AMPK-α/AMPK-α and p-ACC/ACC ratios (Figure 4C). Compound C inhibited the expression level of AMPK-α, p-AMPK-α, ACC and p-ACC and reduced the ratios of p-AMPK-α/AMPK-α and p-ACC/ACC in the T84 cells. Pretreatment with Compound C inhibited the COS-induced modulation of p-AMPK-α/AMPK-α and p-ACC/ACC ratio, indicating that Compound C was effective in inhibiting AMPK activities (Figure 4C). These data suggest that COS modulated the ratio of p-AMPK-α/AMPK-α and p-ACC/ACC both in the presence or absence of afatinib in the T84 cells. In addition, Compound C reversed COS-induced AMPK activation.

### 3.4. Effect of COS on AKT and MAPK-ERK Signaling

**Figure 5:**
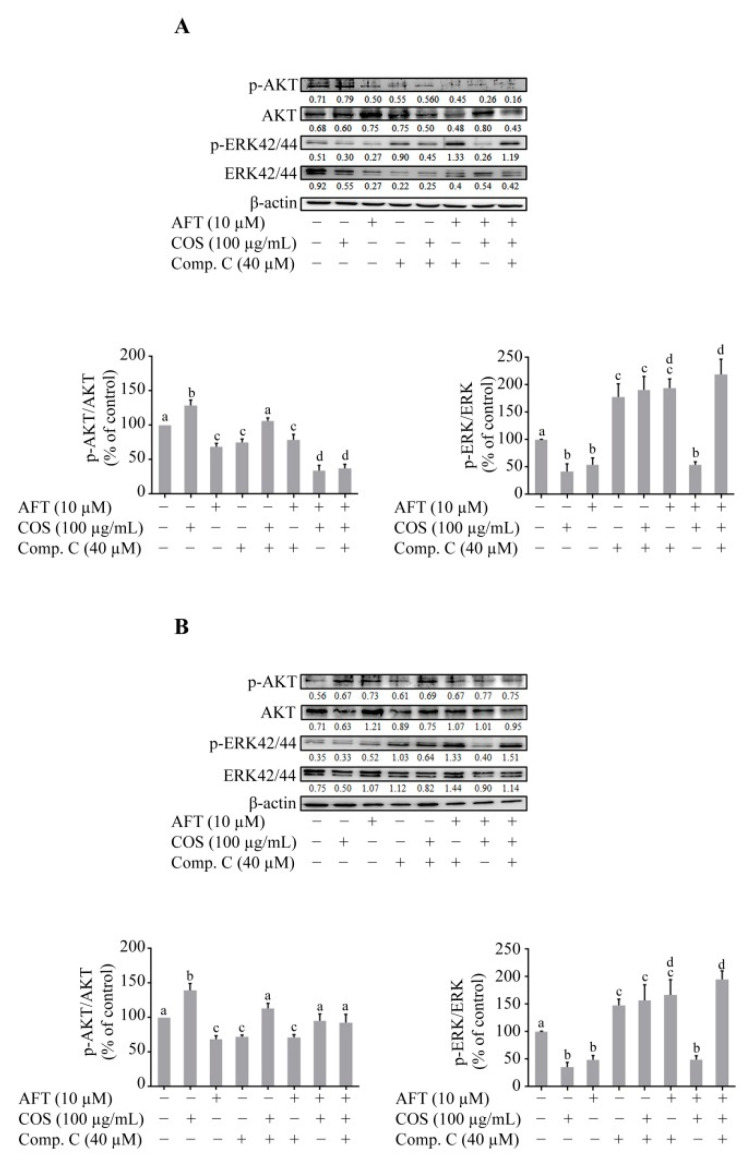
The effect of COS on AKT and ERK phosphorylation. (**A**) T84 cells were treated with the indicated concentrations of COS, afatinib, or both in the presence or absence of Compound C (40 µM) for 24 h. (**B**) In combined treatment, after 30 min pre-incubation of Compound C, cells were exposed to COS for 4 h followed by 24-h afatinib treatment. Data were analyzed as the ratio of p-AKT/AKT, p-ERK/ERK and expressed as % of control (vehicle−treated group), means ± SD (*n* = 3; *p* < 0.05 one-way ANOVA). Columns not sharing the same superscript letters differ significantly (*p* < 0.05, Tukey’s post hoc test).

It is well-established that receptor tyrosine kinases (RTKs) are activated by several growth factors. Upon activation, RTKs activate AKT by phosphorylation at Thr-308 [31,36]. Previous studies have shown that afatinib inhibits the phosphorylation of epidermal growth factor receptor (EGFR) on Tyr-1068 [14,37,38]. We determined the effect of COS and afatinib either alone or in combination in the presence or absence of Compound C on AKT phosphorylation by Western blot analysis. COS significantly induced AKT phosphorylation and increased the ratio of p-AKT/AKT. Afatinib and Compound C, both alone or in combination, inhibited the phosphorylation of AKT and decreased the ratio of p-AKT/AKT in T84 cells, as shown in Figure 5A. Interestingly, COS-induced phosphorylation of AKT was inhibited in the presence of afatinib. Activation of mitogen activated protein kinase (MAPK)/extracellular signal-regulated kinase (ERK) is known to induce the disruption of epithelial tight junctions [39] and MAPK/ERKs are directly regulated by AMPK [39,40]. We determined the effect of COS on ERK phosphorylation in the presence or absence of afatinib and Compound C by Western blot analysis. COS and afatinib significantly inhibited the phosphorylation of ERK1/2 either alone or in combination. Compound C reversed the COS-induced inhibition of ERK1/2 phosphorylation and increased the p-ERK/ERK ratio (Figure 5A).

We next determined the effect of COS pretreatment followed by afatinib treatment in the presence or absence of Compound C on the AKT or ERK pathways. Four-hour pre-incubation with COS increased the expression level of p-AKT and increased the p-AKT/AKT ratio (Figure 5B). Likewise, pretreatment with COS partially reversed the Compound C- and afatinib-induced inhibition of AKT phosphorylation. COS pretreatment produced the same effect and did not alter ERK phosphorylation. Taken together, our results indicated that COS prevented afatinib-induced barrier disruption in T84 cells by modulating the AKT and ERK pathways via both AMPK-dependent and independent mechanisms.

### 3.5. Effect of COS and Afatinib on T84 Cell Viability

**Figure 6:**
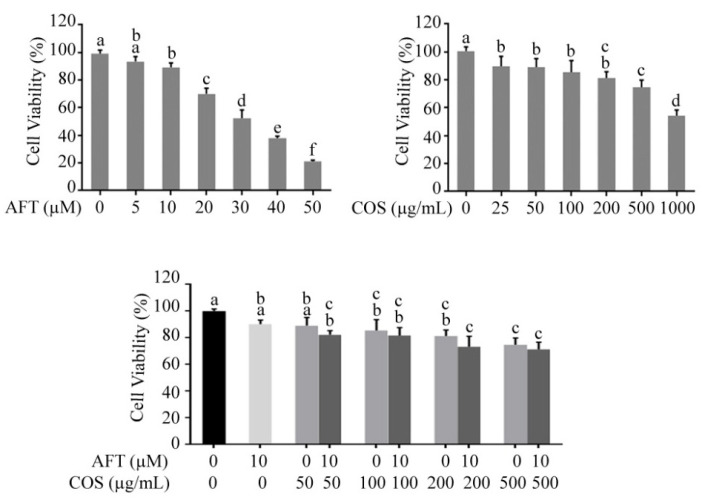
The effect of COS on the anti-cancer activity of afatinib. The effect of afatinib or COS either alone or in combined treatment on the cell viability of T84 cells. Data were expressed as the mean ± SD of five independent experiments. Columns not sharing the same superscript letters differ significantly (*p* < 0.05).

We next determined whether COS affected afatinib-induced cytotoxicity in T84 cells using MTT assays. We found that afatinib and COS inhibited T84 cell viability in concentration-dependent manners (Figure 6A,B). To investigate the effect of COS on the afatinib-induced anticancer effect, T84 cells were treated for 24 h with afatinib (10 µM) combined with COS at various concentrations (50, 100, 200 and 500 µg/mL). We found that COS co-treatment did not affect the cytotoxicity of afatinib in T84 cells (Figure 6C), indicating that COS did not affect the anticancer effect of afatinib.

## 4. Discussion

Unlike conventional chemotherapeutic drugs that induce direct damage to the intestinal epithelium, EGFR-TKIs mediate biological signaling and alter epithelial transport and barrier function. Afatinib, an EGFR-TKI used in this study, has been reported to cause diarrhea by inducing barrier function disruption and calcium-dependent Cl^−^ secretion [14]. In this study, we found that COS protected against the afatinib-induced Cl^−^ secretion in response to forskolin and barrier disruption, at least in part by modulating the AMPK, PI3K/AKT and ERK pathways. In addition, COS had no effect on the anticancer activity of afatinib in the T84 cells.

AMPK, a heterotrimeric protein complex, is composed of α, β, and γ subunits [40]. It is activated by phosphorylation of the AMPK-α subunit at threonine-172 (Thr-172) in response to increased-intracellular Ca^2+^ concentration by calcium/calmodulin-dependent protein kinase kinase β (CaMKKβ) [40,41]. Activated AMPK modulates the functional activities of several proteins involved in energy production and utilization processes, tight junction assembly proteins, and ion transport (i.e., AMPK suppresses CFTR chloride channel activity by phosphorylating at the R-domain) [41,42]. In the present study, COS induced AMPK activation by phosphorylation at Thr-172, characterized by the increased p-AMPK-α/AMPKα ratio. Likewise, COS increased the phosphorylation of ACC, a downstream target protein of AMPK. Furthermore, COS-induced AMPK-α phosphorylation was reversed in the presence of the AMPK inhibitor, Compound C, confirming the inhibitory effect of Compound C on COS-induced AMPK activation.

Upon ligand binding, EGFR is activated, which concomitantly activates the downstream phosphatidyl-inositole-3 kinase (PI3K)/AKT pathway. AKT, a serine/threonine kinase, also known as protein kinase B (PKB), exerts a crucial role in regulating cell proliferation, metabolism, and survival [32]. In intestinal epithelial cells, the tight junction protein is regulated by AKT [32,43]. In addition, the direct activation of EGFR by EGF triggers PI3K/AKT activation, resulting in the inhibition of chloride secretion via an inhibitory effect on basolateral potassium channels [44]. Afatinib induces the reduction in EGFR activation by decreasing its phosphorylation, resulting in the inhibition of AKT phosphorylation [14,36,37,38]. The inhibition of AKT induces chloride secretion, leading to the development of diarrheas. Our data showed that afatinib inhibited AKT phosphorylation and COS partially recuperated the Compound C- and afatinib-induced inhibition of AKT signaling, which could prevent tight junction disruption.

**Figure 7:**
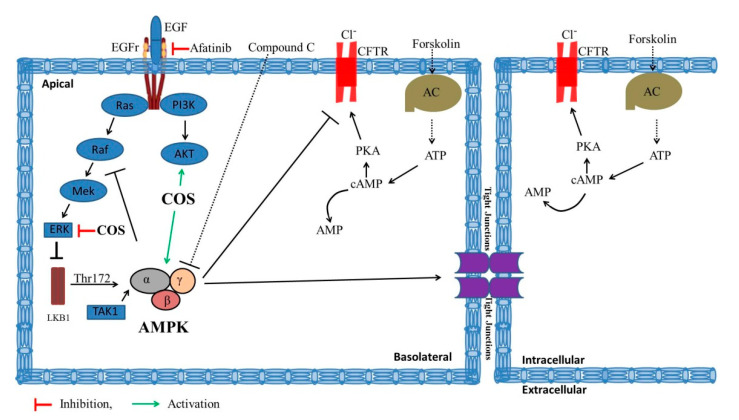
A schematic model summarizing the effect of COS against afatinib-induced intestinal barrier dysfunctions by modulating AMPK, PI3K/AKT, and MAPK/ERK signaling.

MAPK-ERK regulates the cell cycle, gene expression, cell differentiation, cell survival, and apoptosis [45], whereas AMPK regulates cellular metabolism [46]. Several studies have shown that these two signaling cascades have intricate interplays in physiological and pathological processes [40]. MAPK signaling directly regulates AMPK signaling under different conditions. MAPK/ERK phosphorylates and inhibits LKB1, which is an upstream activator of AMPK and thereby blocks the activation of AMPK in BRAF (V600E)-driven melanoma. Likewise, AMPK regulates MAPK signaling. AMPK inhibits the activities of MAPKs by phosphorylation of the RAF/KSR family kinases, which are essential components of MAPKs [40]. The exact behavior of ERK in the regulation of epithelial tight junctions is unknown. Previous studies have revealed that ERK activation may induce or inhibit the disruption of tight junctions in different types of epithelial monolayers [39,47,48]. In one report, ERK activation increased the tight junction assembly by inhibiting claudin-2 expression [47], while, in another report, ERK knockdown increased the tight junction in undifferentiated cell monolayers and exerted an opposite effect in differentiated cell monolayers [39]. Moreover, constitutive activation of Ras or Raf induced ERK activation, which resulted in the disruption of epithelial tight junctions [48]. Our data suggest that COS induces the inhibition of ERK activation and enhances tight junction integrity in T84 cell monolayers. Further investigations are required to delineate the mechanisms by which COS enhances tight junction integrity via ERK inhibition. A schematic model summarizing the effect of COS against afatinib-induced intestinal dysfunctions via modulating AMPK, PI3K/AKT, and MAPK/ERK signaling is shown in Figure 7.

## 5. Conclusions

In conclusion, this study demonstrates that COS represents a potential anti-diarrheal agent for afatinib-associated diarrheas. COS promotes tight junction integrity and prevents afatinib-induced potentiation of cAMP-induced Cl^−^ secretion by activation of AMPK, a positive regulator of epithelial tight junction and a negative regulator of cAMP-induced Cl^−^ secretion, respectively. Furthermore, COS partially reverses afatinib-induced AKT inhibition. Activation of AKT is known to enhance tight junction protein and inhibit Cl^−^ secretion [32,43,44]. COS inhibits MAPK-ERK, indicating that COS might exert an anti-cancer effect. Our findings suggest that COS prevents afatinib-induced Cl^−^ secretion and barrier disruption and enhances tight junction integrity by inducing activation of AMPK and AKT pathways and inhibition of ERK pathways. Due to its action on multiple signaling pathways, further development of COS may provide safe and effective therapies against TKI-associated intestinal toxicities.

## Data Availability

All relevant data are in the manuscript.
